# Milestones in the history of neurocritical care

**DOI:** 10.1186/s42466-023-00271-7

**Published:** 2023-08-10

**Authors:** Rainer Kollmar, Michael De Georgia

**Affiliations:** 1 Department of Neurology and Neurointensive Care, Darmstadt Academic Hospital, Darmstadt, Germany; 2 Department of Neurology, Case Western Reserve University School of Medicine, Cleveland, OH USA

**Keywords:** History, Neurocritical care, Intensive care unit

## Abstract

Over the last century, significant milestones have been achieved in managing critical illness and diagnosing and treating neurological diseases. Building upon these milestones, the field of neurocritical care emerged in the 1980 and 1990 s at the convergence of critical care medicine and acute neurological treatment. This comprehensive review presents a historical account of key developments in neurocritical care in both the United States and Europe, with a special emphasis on German contributions. The scope of the review encompasses: the foundations of neurocritical care, including post-operative units in the 1920s and 30s, respiratory support during the poliomyelitis epidemics in the 40 and 50 s, cardiac and hemodynamic care in the 60 and 70 s, and stroke units in the 80 and 90 s; key innovations including cerebral angiography, computed tomography, and intracranial pressure and multi-modal monitoring; and advances in stroke, traumatic brain injury, cardiac arrest, neuromuscular disorders, meningitis and encephalitis. These advances have revolutionized the management of neurological emergencies, emphasizing interdisciplinary teamwork, evidence-based protocols, and personalized approaches to care.

“Some people want it to happen, some wish it would happen, others make it happen.”

Michael Jordan

## Background

Over the last century, significant milestones have been achieved in managing critical illness and diagnosing and treating neurological diseases. In particular, remarkable advances have been made in treating ischemic stroke and intracerebral hemorrhage, as well as traumatic brain injury, cardiac arrest, neuromuscular disorders, meningitis, and encephalitis. Building upon these milestones, the field of neurocritical care emerged in the 1980 and 1990 s at the convergence of critical care medicine and acute neurological treatment. There is considerable evidence today that treatment in neurocritical care units leads to better outcomes [1, 2].

At the same time, the growth of neurocritical care has given rise to professional organizations dedicated to this field. In 1984, the German Society of Neurocritical Care and Emergency Medicine (DGNI)^1^ was founded and currently has over 1,000 members [3]. In 2023, over 235 intensive care units with specific neurological or neurosurgical expertise are registered with the DGN, while 65 are exclusively neurological and neurosurgical [4]. In Germany, Neurocritical Care is now an officially recognized subspeciality that requires a two-year fellowship in addition to certification in Neurology or Neurosurgery. Neurocritical care has also been incorporated into residency training programs in Germany. More than a third of all emergencies seen in emergency departments in Germany are attributed to neurology and neurosurgery.

In the United States, the Neurocritical Care Society (NCS) was established in 2002 and maintains close professional and academic relations with the DGNI. This comprehensive review presents a historical account of key developments in neurocritical care in both the United States and Europe, with a special emphasis on German contributions.

## Foundations of neurocritical care

The foundations of neurocritical care evolved along with the development of general critical care beginning with the introduction of post-operative units in the 1920s and 30s, advances in respiratory support during the poliomyelitis epidemics in the 40 and 50 s, cardiac and hemodynamic care in the 60 and 70 s, and finally stroke units in the 80 and 90 s.

## Post-operative units in the 1920s and 30s

In 1923, the first three-bed intensive care unit was established at the Johns Hopkins Hospital in Baltimore, Maryland after Walter Dandy, a trainee of neurosurgeon Harvey Cushing, argued that post-op patients should be managed in special units by trained nurses and physicians [5]. Similarly, in the early 1930s, German surgeons Martin Kirschner and Ferdinand Sauerbruch also established so-called “recovery rooms” (“Wachstationen”) where freshly operated patients could receive centralized monitoring [6]. In 1935, Kirschner implemented his vision of a hospital characterized by patient and staff tranquility, optimal illumination, and ventilation, and streamlined hospital navigation.

## Poliomyelitis epidemics in the 1940s and 50s

**Fig. 1:**
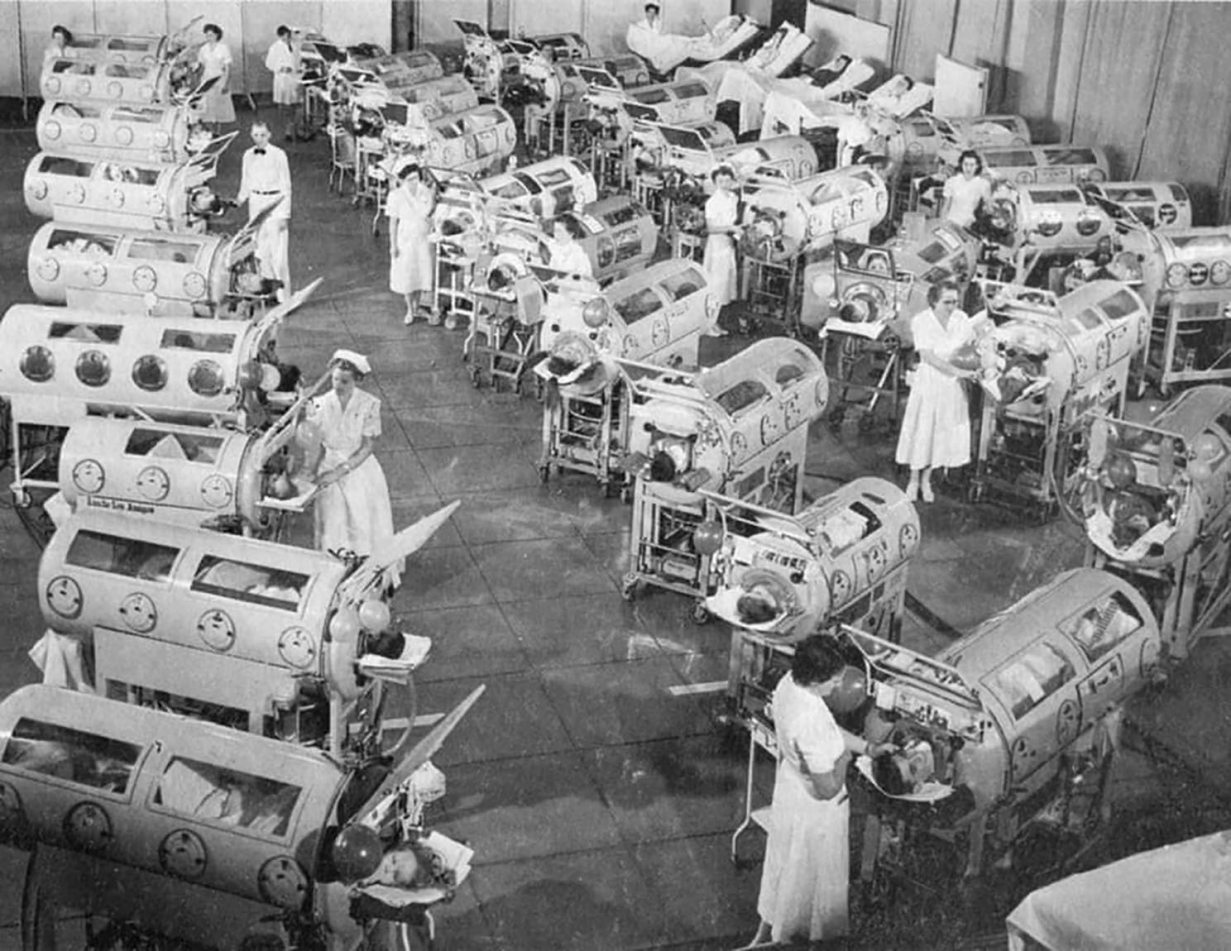
Photograph: Iron lung. Children in iron lungs during a polio outbreak at the Rancho Los Amigos Center in Los Angeles, California in the 1950s. Photograph: Science History Images

The poliomyelitis epidemics of the 1940s and 50s prompted the introduction of vital innovations in respiratory support. Initially, the “iron lung,” a modification of the original tank respirator devised by Scottish physician John Dalziel in 1832, was introduced by Philip Drinker and Louis Shaw from Harvard. Drinker and Shaw’s modification involved electrically operated pumps to create negative pressure, enabling the expansion of the lungs [7]. These “iron lungs” were used throughout the United States during poliomyelitis epidemics [8]. The three largest polio centers were in Rancho Los Amigos in Los Angeles, California (Fig. 1), Boston Children’s Hospital in Boston, Massachusetts, and Cleveland City Hospital in Cleveland, Ohio.

**Fig. 2:**
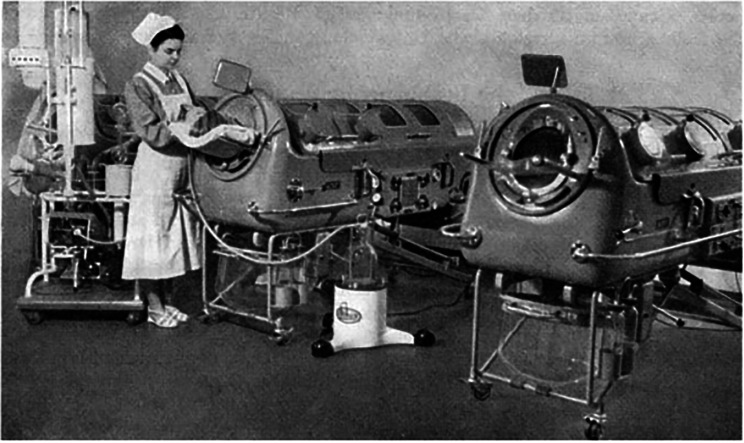
Poliomyelitis ward in Hamburg Altona Hospital with three irons lungs type E52 developed by A. Dönhardt (Dönhardt 1955b)

In Germany, the poliomyelitis epidemics from 1947 to 1952 led to a concentration of polio patients in specific wards in Hamburg Altona (Fig. 2). Because iron lungs could not be imported, Axel Dönhardt, a young fellow under the guidance of Professor Reinhard Aschenbrenner at the University of Hamburg, in collaboration with Deutsche Werft, a ship building company, built new respirators using leftover materials from World War II. These makeshift devices incorporated components from torpedoes, mine detectors, old electromotors, and more [9]. Later, iron lungs would be constructed by the German company Dräger from Lübeck.

In 1948 Dönhardt reported the results of the cerebrospinal fluid (CSF) analysis of 222 polio patients showing that all patients had pleocytosis, low protein, and high glucose levels. He also noted that increased protein content indicated a poorer prognosis [10]. Aschenbrenner and Dönhardt also worked closely alongside Heinrich Pette, a Neurologist from Hamburg, founder of the poliomyelitis research institute in Hamburg (now the Leibnitz Institute for Experimental Virology), and co-founder of the German Neurological Society.

In 1952, because of a limited number of iron lungs in Denmark and a significant portion of patients with bulbar weakness, H.C.A. Lassen, Chief Physician at Blegdam Hospital in Copenhagen, needed a solution quickly. He resorted to emergency tracheostomy and positive pressure bag ventilation, a technique developed by anesthesiologist Bjørn Ibsen, which involved using a soda lime canister to absorb exhaled carbon dioxide [11]. A team of doctors from various specialties and over 250 exhaustingly employed medical students tirelessly provided manual bag ventilation day and night for several weeks. This innovative approach resulted in a remarkable reduction in mortality rates from 80 to 25% [11] and led to the establishment of dedicated respiratory care units all over Europe.

In 1954, the Batten Respiratory Unit in Queens Square, London opened to treat polio patients and those with stroke, spinal cord disorders, and encephalitis. This marked a significant step in respiratory care. That same year, Pierre Mollaret in Paris established the *Centre de Réanimation Respiratoire* at the Claude-Bernard Hospital, further contributing to the advancement of respiratory support [12]. Mechanical ventilation also revolutionized the management of a new category of patients experiencing so-called “fatal” heart attacks.

## Cardiac and hemodynamic care in the 1960s and 70s

Another significant milestone in critical care was the development of defibrillation and resuscitation techniques. In 1947, Claude Beck accomplished the first successful defibrillation of a patient experiencing intraoperative ventricular fibrillation [13]. He later reported the first out-of-hospital resuscitation in 1956 [14]. Both interventions involved thoracotomy, direct cardiac massage, and the placement of defibrillation paddles directly onto the heart.

**Fig. 3:**
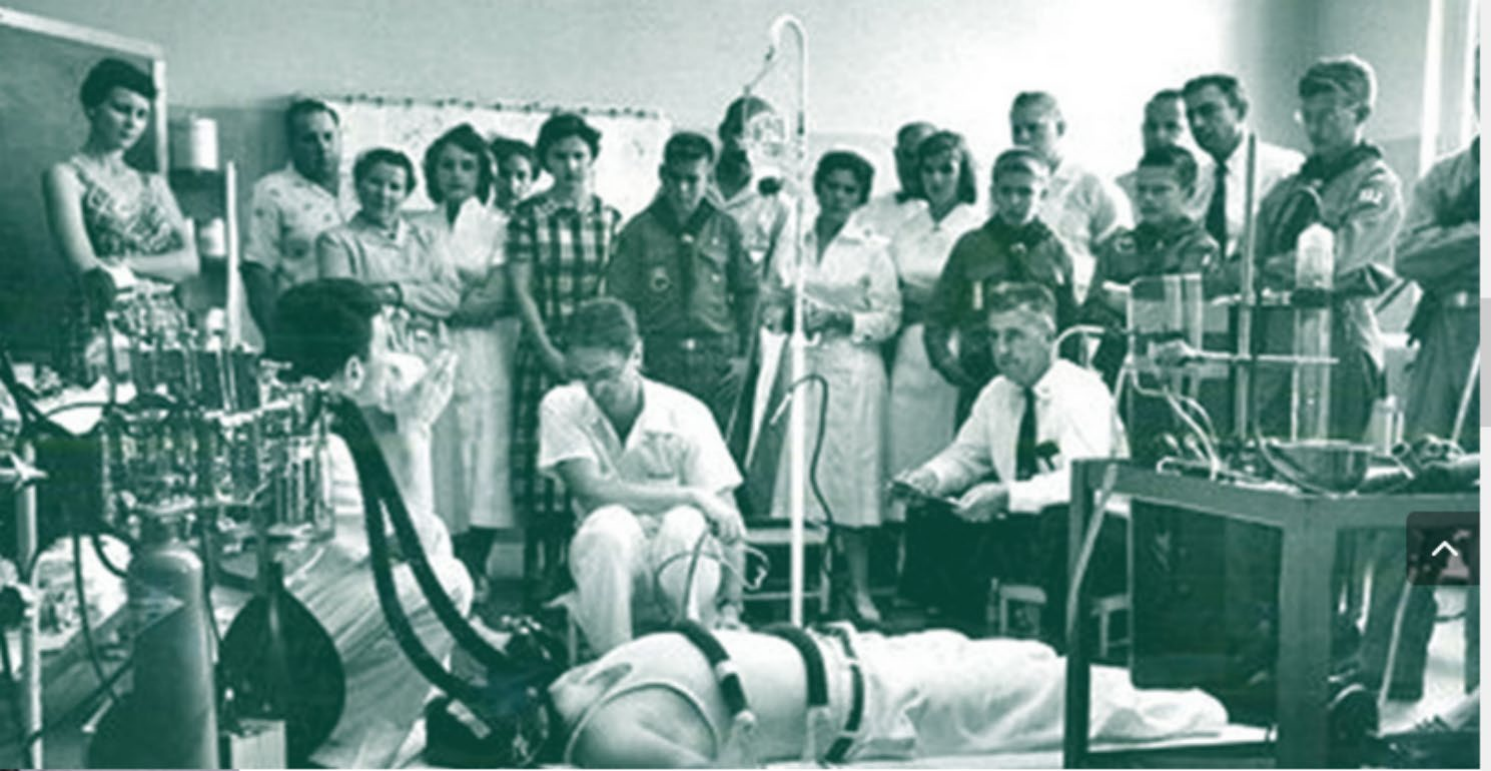
Photo from 1956 of Dr. Peter Safar in the development of the mouth-to-mouth resuscitation component of cardiopulmonary resuscitation (CPR). Dr. Safar is positioned behind the anesthesia machine on the left, managing the airway. Dr. Safar is known as the father of modern-day resuscitation

The first successful human closed-chest defibrillation was reported by Paul Zoll in 1956 [15]. This breakthrough eliminated the need for thoracotomy and direct cardiac access, leading to a less invasive approach. In 1960, William Kouwenhoven introduced closed chest compressions [16]. When combined with mouth-to-mouth breathing, developed by Peter Safar in 1957 [17], the new approach was called “Cardiopulmonary resuscitation” or CPR (Fig. 3).

In 1958, the culmination of these advancements came together when Peter Safar established the first modern “intensive care unit” (ICU) in the United States at Baltimore City Hospital, with a primary focus on airway and respiratory management. Later that same year, Max Weil opened an ICU at the University of Southern California in Los Angeles, initially referred to as a “shock ward,“ which primarily addressed acute circulatory failure. The introduction of continuous electrocardiogram monitoring paved the way for establishing the first Coronary Care Unit (CCU) in 1964 at the Royal Infirmary of Edinburgh in Scotland. The premise was that swift identification and termination of potentially lethal arrhythmias during the peri-infarction period could profoundly alter the course of acute myocardial infarction. This was confirmed in Killip and Kimball’s 1967 landmark article describing a nearly 20% decline in the post-MI mortality rate after implementing their CCU.

The 1960s witnessed further expansion in automated vital signs monitoring with alarms, providing early detection and intervention to prevent physiological deterioration rather than solely treating it after it occurred. Additionally, the introduction of the Swan-Ganz catheter, also known as the pulmonary artery catheter, in 1970 revolutionized patient care [18]. The Swan-Ganz catheter was a great advance, but Swan and Ganz were not the first to perform right heart catheterization. That distinction belongs to a daring 25-year-old surgical intern in Germany named Werner Forssmann at the Auguste-Viktoria Hospital in Eberswalde, who pioneered the technique in 1929 [19]. Forssmann was convinced that right heart catheterization was possible to do. He had practiced it a few times on a cadaver but could not get approval to try it on a patient from his superior, Professor Richard Schneider, Chair of the Department of Surgery. So the industrious young surgeon performed it on himself and then, with the help of a nurse, quickly took an X-ray to prove his point [20]. Forssmann was ultimately awarded the 1956 Nobel Prize in Medicine for this remarkable achievement.

## Stroke units in the 1980s and 90s

Stroke Units served as precursors to modern neurocritical care units. The concept of dedicated, multidisciplinary care for stroke patients emerged in the 1950s in Northern Ireland and the 1960s in the U.S. Initially, these units primarily focused on rehabilitation efforts. In the 1970s, “Stroke Intensive Care Units” were introduced, modeled after the recently opened CCUs, but their impact was inconsistent, partly due to small and heterogenous outcome studies [21]. Nevertheless, the importance of a dedicated team and a focused approach to address stroke complications became increasingly evident. Evidence from a Neuro Vascular Care Unit in San Francisco, predominantly staffed by neurologists, demonstrated fewer complications than conventional ward care [22]. Building upon this insight, Sweden introduced a stroke unit model in the 1980s that integrated acute care with early rehabilitation, leading to reduced mortality rates and improved long-term functional outcomes [23].

A pivotal moment arrived in 1997 when Peter Langhorne from Glasgow, Scotland, representing the Stroke Unit Trialists’ Collaboration, published a seminal systematic review evaluating the effectiveness of organized stroke unit care. This review provided compelling evidence that stroke unit care consistently resulted in sustained reductions in death and dependency, regardless of patients’ age, gender, stroke severity, or variations in stroke unit organization [24]. The essential components of effective stroke unit care included interdisciplinary collaboration, a dedicated focus on reducing complications, evidence-based standardized protocols, and early mobilization and rehabilitation. When these elements were integrated with emerging treatments such as reperfusion therapy, they helped establish the foundation for the development of neurocritical care units.

In the late 90s, some German states established a comprehensive Stroke Unit system, and in parallel, the German Stroke Foundation and German Stroke Society developed a national certification program for hyper-acute stroke units, categorized into three distinct levels of care. Today, there are over 300 certified Stroke Units and Stroke Centers all over Germany. More than 75% of stroke patients receive treatment in certified Stroke Units, with thrombolysis rates at 16% and thrombectomy rates over 7% [25].

## Key innovations

The development of modern neurocritical care relied on three key innovations: cerebral angiography, the computed tomography (CT) scan, and ICP (intracranial pressure) and “multi-modal” monitoring.

## Cerebral angiography

The history of cerebral angiography dates back to 1923, when Berberich and Hirsch performed the first angiography on a hand [26]. In 1927, Portuguese neurologist Egas Moniz introduced cerebral angiography by injecting a sodium iodine solution into the surgically exposed carotid artery [27]^2^. However, the direct carotid puncture required for this procedure carried significant risks and was primarily reserved for cases of suspected aneurysmal subarachnoid hemorrhage. In 1953, Swedish radiologist Sven-Ivar Seldinger developed a technique, now known as the “Seldinger technique,“ which involved using a guidewire to safely thread a catheter into a blood vessel. This advancement made angiography a safer procedure [28].

In 1974, Russian neurosurgeon Fedor Serbinenko reported the technique of balloon embolization and occlusion of the feeding vessels to arteriovenous malformations. He also suggested that cerebral aneurysms could be occluded by injecting coagulating substances to induce clotting and occlusion [29]. These groundbreaking ideas would fundamentally transform the field of vascular neurosurgery. With the evolution of computer technology in the 1980s, digital subtraction angiography (DSA) was developed. DSA allows for the removal of overlapping tissues, providing high-quality images and serving as the gold standard in modern angiography [30].

## Computed tomography

**Fig. 4:**
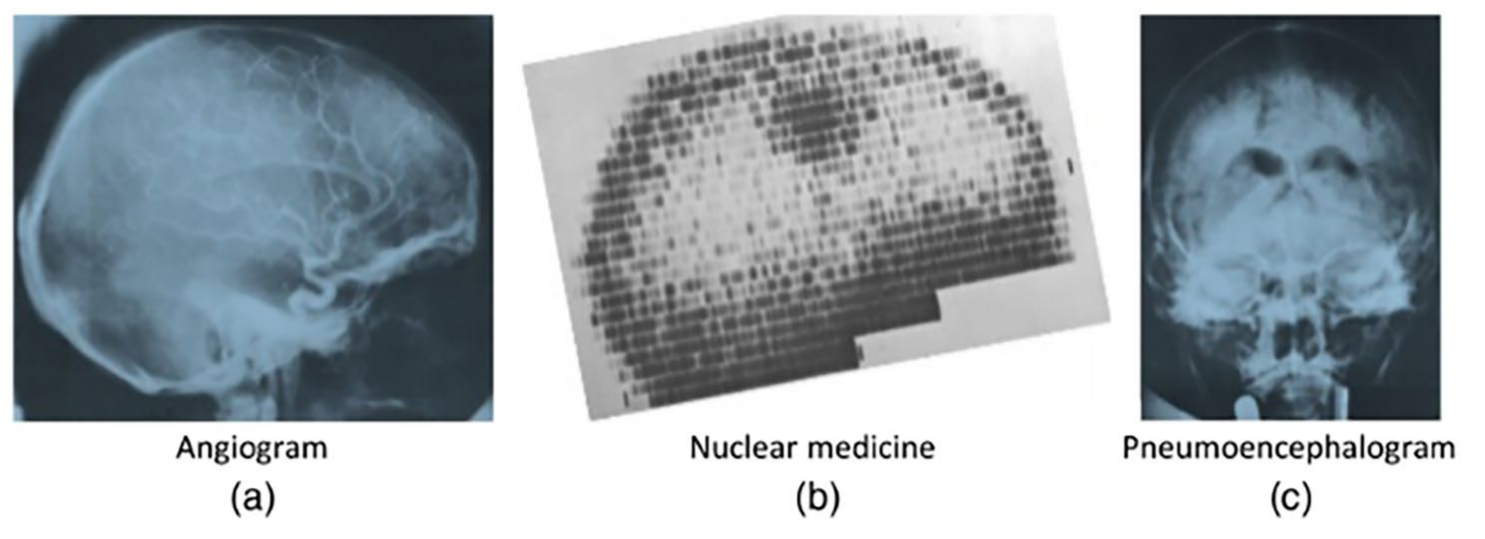
CT replaced numerous invasive procedures in neurology such as angiogram (**a**), nuclear medicine scans (**b**) and pneumoencephalograms (**c**). Photographs are included in the EMI, Ltd., “New EMI machine for diagnosing brain disease,” First EMI Brochure (April 1972)

**Fig. 5:**
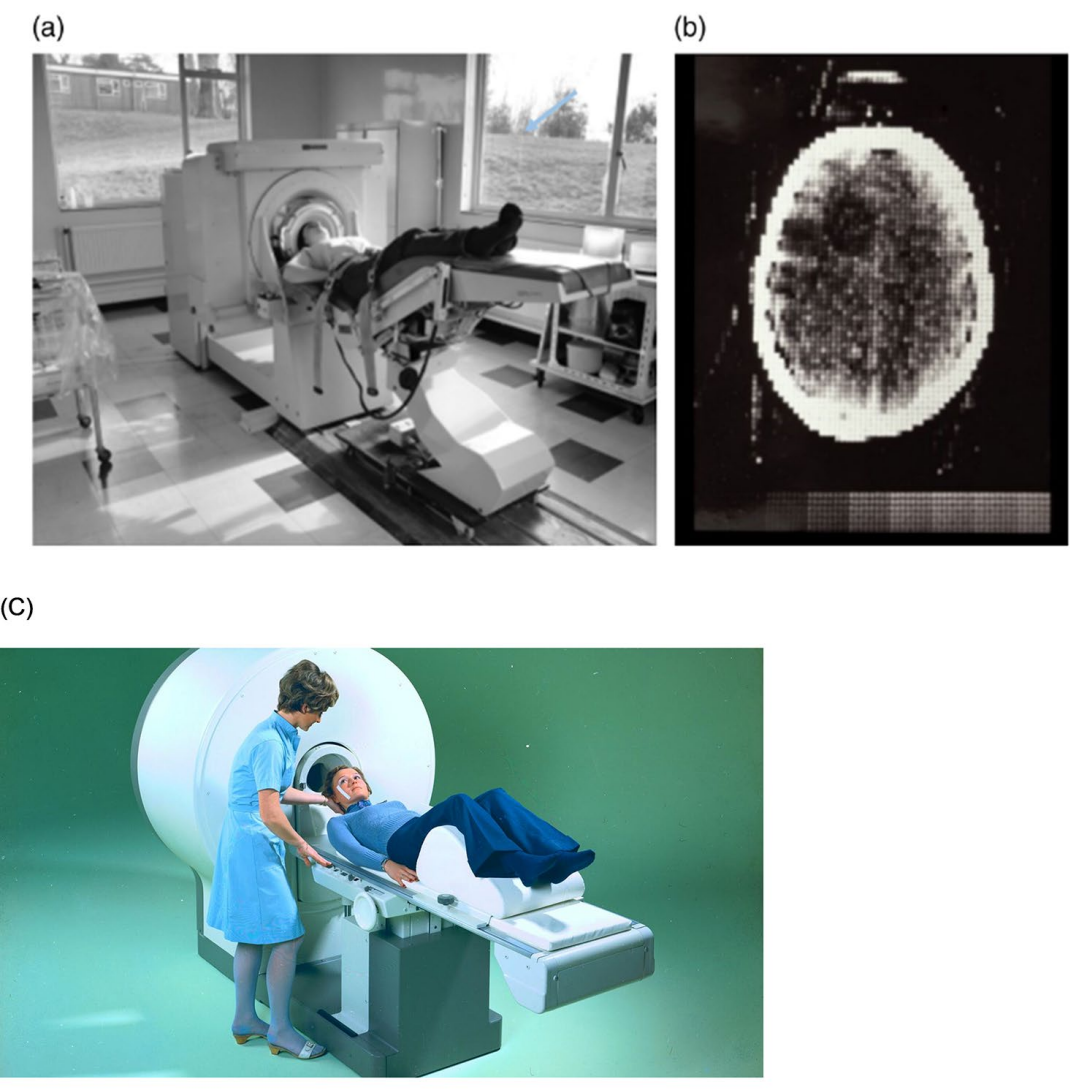
First EMI Scanner (**a**), first CT scan of the brain (**b**), and Siemens prototype CT Scanner Siretom (**c**), Siemens Healthcare GmbH, Erlangen, Germany

The introduction of the CT scan brought about a revolutionary shift in medical practice. It allowed physicians to accurately localize the anatomical location of a lesion and identify the specific cause, enabling more precise and targeted treatments. CT replaced procedures such as angiogram, nuclear medicine scans and pneumoencephalogram (Fig. 4). Developed by British engineer Sir Godfrey Hounsfield and American physicist Allan Cormack in 1968, the first scans took up to nine hours to complete, followed by two and a half more hours of reconstruction before the image could be seen. Hounsfield, employed by the British conglomerate EMI,^3^ introduced the first head CT scanner, the EMI Mark 1, in 1972 [31]^4^. At the same time, a group at Siemens, led by Dr. Friedrich Gudden, was also working on computed tomography independently and without knowledge of Hounsfield’s research at EMI. In 1974, Siemens introduced the SIRETOM CT scanner at Goethe University Hospital in Frankfurt/Main, Germany (Fig. 5). This first model had a single detector and the acquisition of a single slide took more than 5 min [32]. These developments in CT scanning technology revolutionized the field of neurocritical care by providing a non-invasive and accurate method for visualizing and diagnosing intracranial pathologies, facilitating prompt and appropriate interventions.

More recently, the development of advanced computer algorithms has enabled the high-resolution visualization of cerebral vasculature through contrast injection, known as CT angiography. Simultaneously, perfusion algorithms have been developed to identify a “mismatch” between hypoperfused yet salvageable brain tissue and irreversibly infarcted tissue [33]. This concept of “mismatch” has revolutionized stroke care by identifying patients who may benefit from reperfusion therapy.

## Intracranial pressure and multi-modal monitoring

**Fig. 6:**
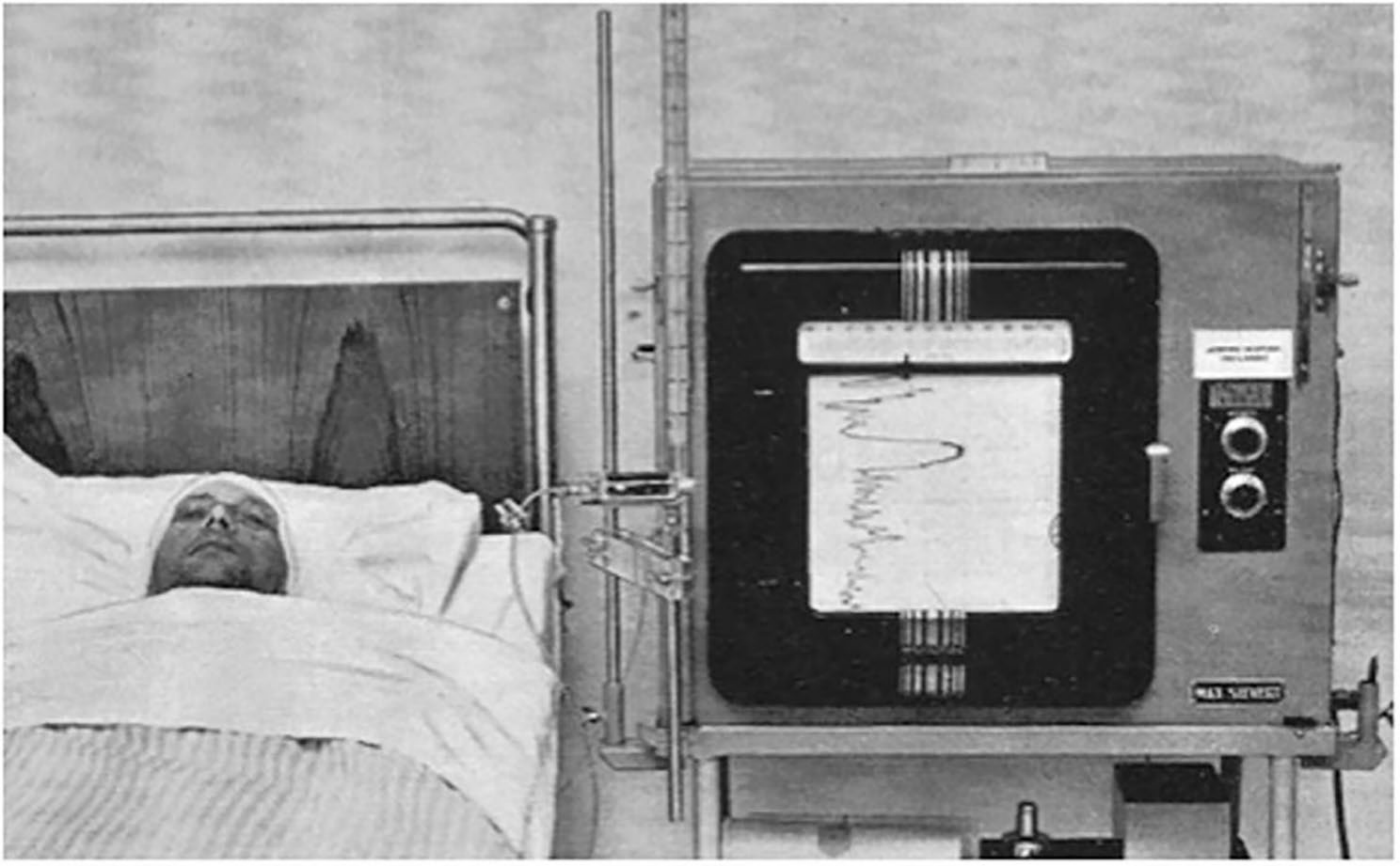
Image from Lundberg’s 1960 publication on continuous recording and control of ventricular fluid pressure in neurosurgical practice

Intracranial pressure monitoring represented a major innovation, especially in managing patients with traumatic brain injury (TBI) and intraventricular hemorrhage. It was the Swedish neurosurgeon Nils Lundberg who first introduced ICP monitoring in 1965 [34], (Fig. 6) Lundberg had developed the technique in the 1950s at Lund University Hospital as part of his doctoral thesis [35].

In 1975, Bryan Jennett, a neurosurgeon in Glasgow, Scotland, observed that many patients with head injury seemingly “talked and died,” suggesting that additional factors other than their primary injury were important [36]. Recognition of this “secondary brain injury,” from ischemia, tissue hypoxia, and a cascade of metabolic events, translated into the need for aggressive critical care to avoid or rapidly correct adverse factors. In the 1990s and early 2000s, prevention of secondary brain injury spurred development of new neuromonitoring tools, including those for cerebral perfusion, brain tissue oxygenation, and brain metabolism. Taken together with ICP monitoring, this approach became known as “multi-modal monitoring” [37]. Though there is insufficient evidence regarding the impact of multi-modal monitoring on clinical outcome, ICP monitoring remains the cornerstone of monitoring in neurocritical care [38].

## Advances in ischemic stroke and intracerebral hemorrhage

### Ischemic stroke

#### Thrombolysis

Acute ischemic stroke currently represents the largest cohort in the field of neurology, with its incidence and prevalence on the rise [39]. Advances in treating acute ischemic stroke in the 1980s and 90s played a pivotal role in the development of neurocritical care. No history of stroke, however, would be complete without recognizing the pivotal 1846 publication by Rudolf Virchow describing the pathophysiology of arterial thrombosis [40]. This laid the foundation for understanding the underlying mechanisms of acute myocardial infarction and ischemic stroke.

A revolution in thrombolysis for acute myocardial infarction began in the 1980s. In 1985, the Western Washington Study was the first large randomized trial of intra-arterial thrombolysis using streptokinase for acute myocardial infarction. It demonstrated successful recanalization of occluded coronary arteries, leading to improved one-year survival rates [41]. The following year, the Italian Group for the Study of Streptokinase in Myocardial Infarction (GISSI) published the first large randomized trial of intravenous thrombolysis for myocardial infarction, demonstrating that it was just as effective as intra-arterial thrombolysis when administered up to six hours after symptom onset [42].

Concurrently, a parallel revolution in thrombolysis for acute ischemic stroke was driven largely because of availability of Neurocritical Care Units. In Aachen at the RWTH Technical University, Neurologist and Neuroradiologist Hermann Zeumer and Werner Hacke, the attending neurologist of the Neurocritical Care Unit, guided by their mentor Klaus Poeck, pioneered the field of intra-arterial thrombolysis for stroke. They published the first paper on intra-arterial thrombolysis in acute basilar artery occlusion in 1982 [43], followed by a subsequent publication summarizing their experience with the initial four cases of intra-arterial recanalization in acute stroke a year later [44]. From 1983 to 1986, Hacke and Zeumer collaborated with hematologist Greg del Zoppo at the Scripps Clinic and Research Foundation in La Jolla, California on a pilot trial of intra-arterial thrombolysis in patients with acute middle cerebral artery (MCA) occlusion [45] and a retrospective review of intra-arterial thrombolysis for vertebrobasilar occlusive disease [46]. In 1986, Hacke was a visiting professor at the Scripps Clinic and a year later, he became Chair of the Department of Neurology at the University of Heidelberg’s newly opened “Kopfklink.”

In 1990, Hacke hosted the first International Symposium on Thrombolytic Therapy in Acute Ischemic Stroke, leading to the publication of the proceedings in 1991 [47]. As evidence mounted favoring recombinant tissue plasminogen activator (rt-PA) as a more effective thrombolytic agent, Hacke and von Kummer published the first case series on rt-PA use for acute ischemic stroke [48]. That same year, a German – American collaboration led to an open angiography-based dose escalation study that demonstrated the effect of intravenous rt-PA on recanalization in patients with acute ischemic stroke [49]. The Global Utilization of Streptokinase and Tissue Plasminogen Activator for Occluded Coronary Arteries (GUSTO) trial in 1993 confirmed the superiority of rt-PA, particularly when administered with a bolus, over streptokinase for occluded coronary arteries within a six-hour time window [50].

Hacke’s significant contribution continued in 1995 with the publication of the European Cooperative Acute Stroke Study (ECASS), the first large randomized controlled trial to examine the efficacy of intravenous rt-PA for acute ischemic stroke using a six-hour time window and rt-PA dose of 1.1 mg/kg [51]. No significant differences between the groups were identified in the intention-to-treat analysis, however, a target population analysis (removing protocol violations) favored rt-PA. The NINDS-sponsored trial, using a shorter time window (three hours) and a smaller dose (0.9 mg/kg), demonstrated a better functional outcome at three months [52]. These findings led to the regulatory approval of rt-PA in the United States in 1996 and Germany in 2000.

In 1999, Furlan and colleagues demonstrated the effectiveness of intra-arterial thrombolysis for MCA occlusion in the Pro-urokinase in Acute Cerebral Thromboembolism PROACT II study [53], laying the foundation for mechanical thrombectomy. ECASS III, led by Hacke in 2008, extended the time window for intravenous rt-PA administration to 4.5 h for selected patients [54]. More recent studies have shown that a subset of patients who would have previously been considered outside the time window for rt-PA treatment, such as those with “wake-up strokes,“ may still benefit from treatment when identified with restricted diffusion lesions on magnetic resonance imaging but without corresponding changes on fluid-attenuated inversion recovery sequences [55]. A meta-analysis of individual patient data from the randomized controlled trials EXTEND, ECASS4-EXTEND, and EPITHET indicated that patients with salvageable brain tissue based on penumbral imaging benefit from intravenous thrombolysis within a time window of 4.5 to 9 h from stroke symptom onset [56].

### Mechanical thrombectomy

In the early 2000s, it was recognized that mechanical retrieval of a thrombus, in combination with intravenous rt-PA, might be more effective than intra-arterial thrombolysis. The Merci Retrieval System was the first device approved for intra-arterial clot extraction by the FDA in 2004 [57]. Second-generation thrombectomy devices using clot aspiration received FDA approval in 2007. A newer generation of thrombectomy retrieval devices, employing a stent-based design to entrap the clot, gained FDA approval in 2012. Stent retrievers, known for their higher recanalization rates and shorter procedure times, were expected to yield more favorable functional outcomes. In 2015, 75 years after Moniz’s paper, five randomized trials showed the efficacy of mechanical thrombectomy over standard medical care in patients with acute ischemic stroke caused by occlusion of arteries of the proximal anterior circulation [58]. Based on multimodal penumbral imaging, the time window for recanalization is now personalized rather than restricted by fixed periods from stroke symptom onset [59].

These advancements in thrombolysis and thrombectomy have significantly transformed the management of acute ischemic stroke, allowing for more effective and timely interventions and improving patient outcomes in neurocritical care.

### Decompressive craniectomy

**Fig. 7:**
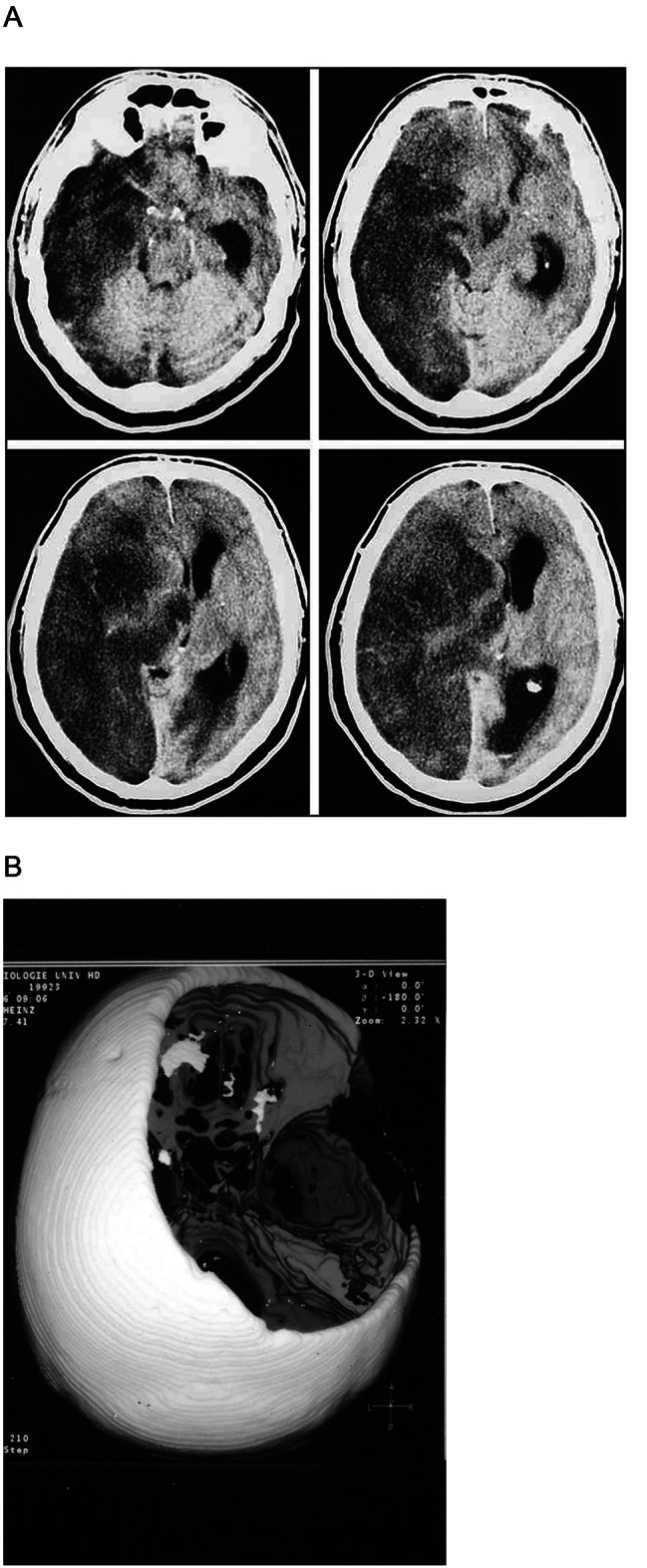
(**A**) From the original publication of Hacke et al. [60] (52). Non-contrast computed tomographic scans of a 56-year-old man 34 h after onset of severe right-sided hemiparesis. The midline shift was measured with a ruler on the individual images and converted to the true dimensions; the midline shift was 12 mm. The patient had a unilateral fixed, dilated pupil. He died 12 h later. (**B**) 3-dimensional model of hemicraniectomy indicating the necessary size for bone removal

Intracranial hypertension often occurs when there is significant swelling in severe TBI, large ischemic stroke, or intracranial hemorrhage. Decompressive craniectomy is a highly effective method for controlling intracranial pressure by creating more space for the injured brain. During the 1990s in Heidelberg, decompressive craniectomy was introduced as potential treatment option for patients with large infarcts complicated by cerebral edema and midline shift, known as “malignant” middle cerebral artery (MCA) - territory strokes. While there were case reports and small case series in the 1980s and early 1990s, standard management had been essentially extrapolated from TBI, relying heavily on osmotic agents to address elevated intracranial pressure. Hacke and coworkers described the natural history of malignant MCA-territory strokes and showed that the mortality was around 80% with standard medical treatment [60], (Fig. 7). Klaus Rieke and Stefan Schwab from Heidelberg also published the first case series on decompressive surgery for these patients, with 32 treated with hemicraniectomy and 21 treated with maximal medical therapy. The survival rate was 66% in the surgery group vs. 24% in the medical group [61].

These pilot studies in Heidelberg provided preliminary evidence for the potential benefit of early decompressive craniectomy in patients with MCA-territory stroke, setting the stage for a larger randomized trial [62]. The German DESTINY trial demonstrated a significant reduction in one-year mortality, with rates of 53% in the conservative treatment group versus 18% in the surgery group [63]. A pooled analysis of other trials, published in 2007, demonstrated a similarly dramatic reduction in mortality with hemicraniectomy (78% vs. 29%) along with improved functional outcomes at one year [64]. While the initial randomized controlled trials were restricted to patients younger than 60 years old, the DESTINY II trial demonstrated that decompressive craniectomy also reduces mortality in patients older than 60 years old by close to 50%. Functional outcomes, while improved, were not as good as in younger patients [65]. Based on these trials, decompressive hemicraniectomy has fundamentally changed how patients with malignant hemispheric strokes are managed.

### Intracerebral hemorrhage

#### Blood pressure reduction

Intracerebral hemorrhage, another life-threatening condition, has also seen advances in treatment strategies. Early data from the 1990s highlighted the correlation between hemorrhage volume and clinical outcome [66]. Several trials demonstrated the efficacy of early aggressive blood pressure reduction and anticoagulation reversal in improving outcomes [67]. Based on the results from the second Intensive Blood Pressure Reduction in Acute Cerebral Hemorrhage Trial (INTERACT-2) [68] and the Antihypertensive Treatment of Acute Cerebral Hemorrhage-II (ATACH-2) trial [69], current guidelines recommend targeting a systolic blood pressure between 130 and 150 mm Hg [70]. Recently, results of the INTERACT 3 trial showed that, in addition to early intensive lowering of systolic blood pressure, an acute care bundle protocol that includes strict glucose control, fever prevention, and rapid reversal of anticoagulation also significantly improves functional outcome [71].

### Minimally invasive surgery and intraventricular thrombolysis

After conventional craniotomy and hematoma evacuation yielded negative results in the Surgical Treatment for Intracerebral Hemorrhage (STICH) trials [72, 73], there was a renewed interest in using minimally invasive surgery. Ludwig Auer, an Austrian neurosurgeon, reported the first randomized trial of endoscopic hematoma evacuation in 1989, showing a lower mortality rate with evacuation [74]. In 2016, Dan Hanley from Johns Hopkins Hospital in Baltimore, Maryland published the results of the Intraoperative Stereotactic Computed Tomography–guided Endoscopic Surgery for Brain Hemorrhage (ICES) trial. Compared with the medical arm from the Minimally Invasive Surgery plus Alteplase in Intracerebral Hemorrhage Evacuation (MISTIE) trial, the surgical group showed a trend towards a favorable outcome [75]. Several large-phase trials are currently underway, and a recent meta-analysis of randomized trials has shown that select patients benefit from minimally invasive clot removal [76].

Targeting intraventricular hemorrhage has also shown promise in improving outcomes. Collaborating with Hacke in Heidelberg, Hanley reported a case series in 1996 of patients receiving intraventricular rt-PA, which resulted in faster clearance of intraventricular blood [77]. In 2017, Hanley published the results of the Clot Lysis Evaluation of Accelerated Resolution of Intraventricular Hemorrhage (CLEAR-III) trial, comparing intraventricular rt-PA injection to placebo. The treatment group demonstrated a 10% reduction in mortality and improved functional outcomes for patients with larger initial intraventricular hemorrhage volumes [78].

### Spectrum of conditions requiring neurocritical care

#### Cardiac arrest

**Fig. 8:**
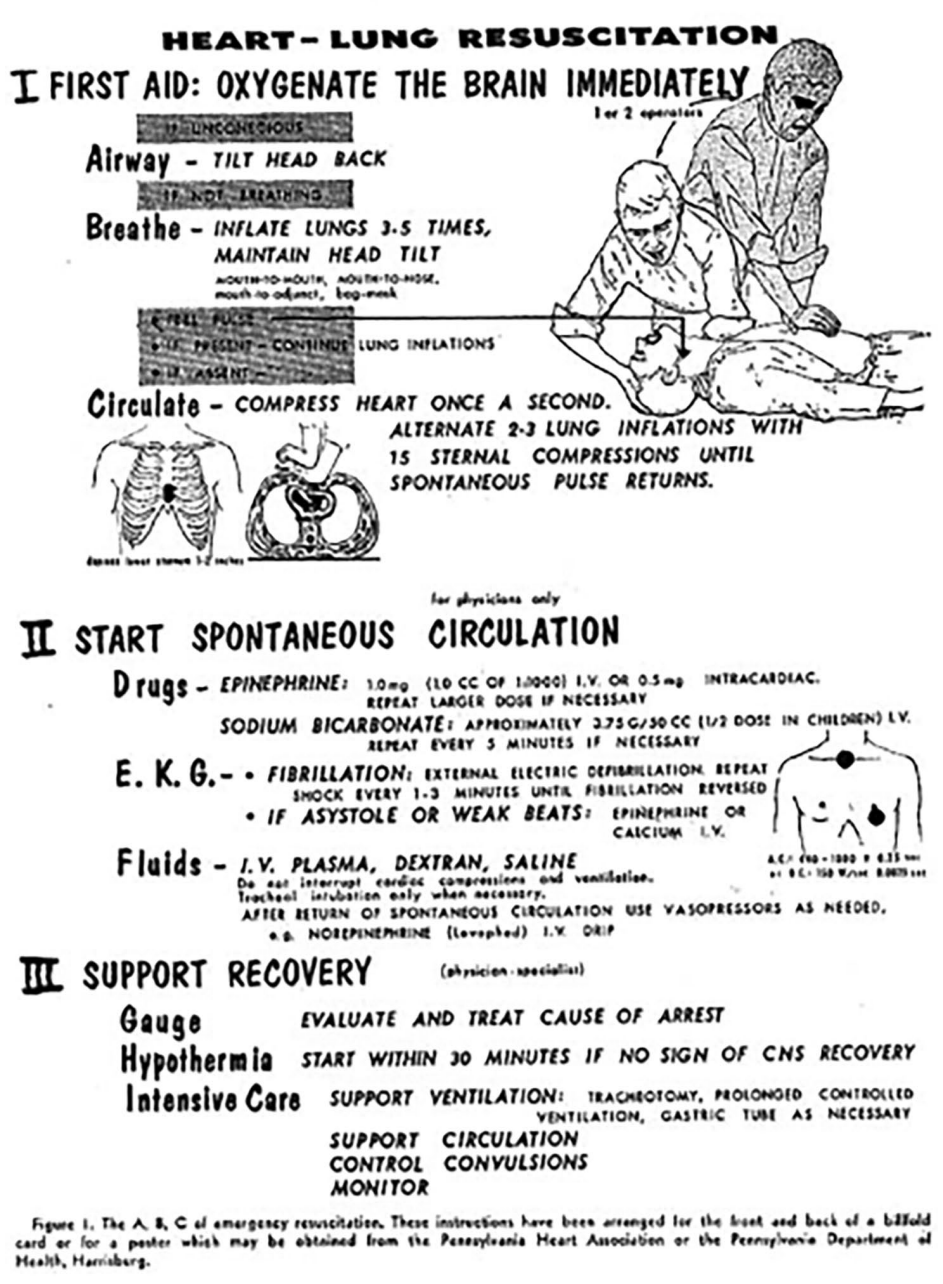
Initial description of the ABCs of resuscitation by Dr. Peter Safar in this classic publication in the Journal of the Iowa Medical Society [79]

Despite improvements in resuscitation following cardiac arrest, among survivors, anoxic brain injury remains the most devasting consequence. In 1964, Peter Safar advocated for therapeutic hypothermia as a potential intervention to mitigate neuronal damage to be incorporated into the emerging treatment protocols, the “ABCs” of resuscitation [79], (Fig. 8) In the 1990s, animal models of cardiac arrest (particularly clinically relevant dog models developed in Safar’s lab at the University of Pittsburgh [80]) confirmed improved functional recovery and reduced cerebral histologic deficits with therapeutic hypothermia [81]. In 2002, the Hypothermia After Cardiac Arrest trial (led by Fritz Sterz, a former fellow of Safar’s) demonstrated that therapeutic hypothermia could significantly reduce mortality and improve neurologic outcomes [82]. While the results of recent European trials (TTM1 and TTM2) have challenged the benefits of induced hypothermia, “Targeted Temperature Management” post-cardiac arrest remains the standard of care [83]. Therapeutic hypothermia has not been shown to be effective in improving outcomes in ischemic stroke or traumatic brain injury.

### Traumatic brain injury

Traumatic brain injury management also evolved in parallel with advances in critical care, neurosurgical techniques, brain imaging, and ICP monitoring. A key breakthrough came in 1960 when Lund introduced ventricular catheters, enabling the therapeutic drainage of CSF to be integrated into standard treatments for intracranial hypertension alongside osmotic agents and hyperventilation [34]. Subsequently, in 1971, bifrontal decompressive craniectomy was introduced to address refractory elevated ICP [84]. The pivotal work of Miller and Becker in 1977 demonstrated improved outcomes by incorporating ICP monitoring into clearly defined treatment algorithms [85, 86].

Implementing craniotomy and mass lesion evacuation also played a crucial role in reducing mortality associated with TBI. Archaeological findings trace the practice of craniotomy back to at least 5000 BC in Europe [87], while the Italian physician Berengario da Carpi documented three cases of craniotomy for TBI in 1518 [88]. During World War I, Harvey Cushing’s introduction of subtemporal craniectomy for brain decompression decreased the mortality rate of severe TBI to 35% [89]. However, the effectiveness of this procedure was often limited by the absence of aseptic techniques, leading to severe infections. With the advent of antibiotics and better surgical techniques during World War II, the mortality rate dropped to approximately 14% [90]. The introduction of the CT scan would revolutionize neurocritical care by allowing quick identification of mass lesions. In 1981, combining CT scanning, early operative decompression, and ICP monitoring, Seelig and colleagues, in a landmark paper, demonstrated that early evacuation of subdural hematomas (within four hours) cut the mortality rate from 90 to 30% [91].

Even with early surgery and aggressive intensive care, however, many patients did not survive, and accurate prognostication remained challenging. In 1974, neurosurgeons Graham Teasdale and Bryan Jennett developed the Glasgow Coma Scale (GCS), which incorporated assessments of the best eye response, verbal response, and motor response [92]. The GCS later became incorporated into Advanced Trauma Life Support training and the National Traumatic Coma Databank guidelines [93]. To address limitations in sedated and intubated patients, Eelco Wijdicks from the Mayo Clinic introduced the Full Outline of UnResponsiveness (FOUR) score in 2005. By encompassing evaluations of eye response, motor response, brainstem reflexes, and respiration, the FOUR score offers a more accurate prediction of outcomes than the GCS score [94].

### Status epilepticus

Status epilepticus is a life-threatening neurological emergency common in the neurocritical care unit with a mortality rate of around 20% [95]. Although prolonged seizures have been recognized since ancient times (the earliest recorded description dating back to about 700 BC), a comprehensive understanding of its pathophysiology and effective treatment has only recently been achieved. Various remedies in the nineteenth century included cupping glasses, bloodletting, ice bags, quinine, ammonia inhalation, potassium bromide, and opiates. The first effective treatment, amyl nitrate, was reported by Wildermuth from Stuttgart in 1889 [96]. Subsequently, barbiturates were introduced in 1912, followed by phenytoin in 1937 [5].

The modern definition of status epilepticus emerged from the 10th European Electroencephalographic Meeting (the Marseilles Colloquium) in 1962 when Gastaut and colleagues proposed that status be defined as “a condition characterized by an epileptic seizure which is so frequently repeated or so prolonged as to create a fixed and lasting epileptic condition” [97]. Three years later, Gestaut introduced the use of benzodiazepines [98]. The Veterans Affairs Cooperative Trial was a landmark study that confirmed the value of lorazepam as the first agent for the treating status [99]. Benzodiazepines should be used first, followed by levetiracetam, valproic acid, or fosphenytoin if the seizures have not stopped. According to the Established Status Epilepticus Treatment Trial (ESETT), all three antiepileptic medications have similar treatment efficacy [100]. However, in up to 43% of patients, status epilepticus is still not controlled. This so-called “refractory status epilepticus” requires intravenous anesthetic drugs such as midazolam, propofol, ketamine, volatile anesthetics, or a ketogenic diet [101].

### Neuromuscular disorders

Neuromuscular disorders, such as Guillain-Barré syndrome (GBS) and Myasthenia Gravis (MG), though relatively uncommon, frequently give rise to respiratory failure, necessitating the use of ventilatory support. GBS was first described by Guillain, Barré, and Strohl in two French soldiers in World War I [102]. They observed high CSF protein levels without inflammatory white cells — their socalled “dissociation albumino-cytologique.” This finding was distinct from the high white cell counts seen in the CSF of patients with other prevalent causes of acute flaccid paralysis, such as syphilis or polio. It took many years, however, to characterize pathophysiological processes and develop effective treatment strategies [103]. The cause is from immune-mediated demyelination, axonal damage, or both of peripheral nerves due to molecular mimicry between microbial antigens (e.g., Campylobacter jejuni, Mycoplasma pneumonia, cytomegalovirus, Epstein-Barr virus, influenza virus) and neuronal antigens [104]. In 1985, a pivotal North American trial demonstrated that plasma exchange in patients who couldn’t walk hastened recovery, especially when started within two weeks of onset [105]. Based on the beneficial effects observed in chronic inflammatory demyelinating polyneuropathy, intravenous immunoglobulin (IVIG) was proven as effective as plasma exchange in a Dutch trial in 1992 [106].

Wilhelm Erb of Heidelberg was the first to describe the most important characteristics of Myasthenia gravis, namely oculobulbar symptoms and fluctuating remissions and exacerbations. Friedrich Jolly of Berlin coined the term “myasthenia gravis pseudoparalytica” [107]. In the 1930s, the discovery of physostigmine as the first acetylcholinesterase inhibitor marked a significant milestone, subsequently replaced by pyridostigmine and neostigmine as first-line agents for treating myasthenia gravis [108].

In 1977, Klaus Toyka of Würzburg published a seminal paper demonstrating that myasthenia gravis involves an antibody-mediated autoimmune attack on the acetylcholine receptors at the neuromuscular junction [109], leading to the development of immunosuppressant treatment strategies such as corticosteroids, azathioprine, mycophenolate mofetil, methotrexate, cyclosporine, tacrolimus, cyclophosphamide, IVIG, and plasma exchange. Wolfgang Müllges established plasma exchange as a therapy for myasthenic crisis [110]. In neurocritical care, a combination of acetylcholinesterase inhibitors, corticosteroids, and IVIG or plasma exchange is often needed to manage patients. Advancements in immunology have given rise to novel therapeutic agents and techniques, including complement inhibitors, FcRn inhibitors, B cell inhibitors, T cell inhibitors, and even hematopoietic stem cell transplantation [111].

### Meningitis and encephalitis

Bacterial meningitis represents a life-threatening infectious disease with a high mortality of 20–30%. Treatment with antibiotics goes back to the use of sulfonamides by Francois Schwentker and penicillin by Chester Keefer [112]. The European clinical multicenter study on the effects of dexamethasone treatment prior to antibiotics, carried out in neurocritical care units, demonstrated a better neurological outcome and experience fewer systemic complications with steroids [113].

A large proportion of meningitis is of viral origin, with herpes simplex viruses (HSV) being common. Mortality rates before effective antiviral therapy were as high as 70% [114]. Even with antiviral treatment, only 38% of patients return to their premorbid level of function. When HSV encephalitis is suspected, empiric antiviral treatment should be started as soon as possible. A delay of two days leads to a three-fold increase in poor outcome at six months [115]. Complications of HSV Encephalitis include seizures, cerebral edema, and brain herniation. The German trial on Aciclovir and Corticosteroids in Herpes-simplex-virus-Encephalitis (GACHE), initiated by German neurointensivists, was unfortunately stopped due to slow recruitment [116]. Thus, the use of steroids in addition to antiviral treatment remains experimental.

A decade ago, post-infectious autoimmune encephalitis was discovered, namely Anti-N-methyl-D-aspartate receptor (NMDAR) encephalitis following HSE was first reported by Prüss and colleagues from the Charité Hospital in Berlin [117]. Anti-NMDAR antibodies of the immunoglobulin subtypes IgA, IgG, or IgM were detected in 30% of patients during HSE, suggesting secondary autoimmune mechanisms. The presence of these antibodies is associated with classic autoimmune encephalitis in most patients, though notably some patients who developed anti-NMDAR antibodies after HSE did not clinically develop autoimmune encephalitis [118]. Typically, patients with later develop autoimmune encephalitis have symptoms within the first two months after HSV encephalitis, however, a delayed onset up to a year has been reported [118]. First-line treatment includes steroids, IVIG, and plasma exchange followed by rituximab, cyclophosphamide, and steroid-sparing agents in the long term.

### Coma and brain death

Advances in critical care, resuscitation, and cardiopulmonary support in the 1960s created unique diagnostic, prognostic, and ethical dilemmas for physicians. Distinguishing patients with severe but potentially recoverable brain injuries from those “hopelessly unconscious” was challenging. In 1968, the Ad Hoc Committee of the Harvard Medical School published its definition of Irreversible Coma and established standard criteria for “Brain Death” [119]. In Germany, discussions about the determination of brain death started the same year [120, 121]. Over the next several decades, neurologists refined the understanding of brain death and created clear guidelines for withdrawal of care and organ donation [122]. In Germany, diagnostic procedures for the diagnosis of irreversible loss of brain function (“Hirntoddiagnostik”) have been regularly updated by the German Medical Association since 1982 [123].

### The birth of neurocritical care

The field of neurocritical care emerged due to the convergence of critical care medicine and advances in diagnosing and treating acute neurological illness. Europe, and especially Germany, played a significant role in its development. In Germany, critical care in the 1960s and 70s evolved mainly as part of anesthesiology, leading the German Society for Anesthesiology in 1977 to change its name to the German Society for Anesthesiology and Intensive Medicine. Neurosurgery ICUs were mainly staffed following this model [124]. However, other medical specialties were free to create their own intensive care units [125]. By 1975, more than 15 Neurology departments had opened either separate neurology intensive care units or combined units with neurosurgery.

In 1984, a pivotal event occurred when Werner Hacke published *Neurologische Intensivmedizin*, one of the first books dedicated to the emerging specialty [126]. In the same year, the German Society of Neurocritical Care and Emergency Medicine was founded. A decade later, Hacke published *NeuroCritical Care*, a collaborative textbook that brought together German and American authors (Allan Ropper from Massachusetts General Hospital served as guest editor and Dan Hanley as one of the co-editors) [127]. In the U.S., the Neurocritical Care Society was established in 2002. At the inaugural NCS meeting in 2003, Hacke, Ropper and Hanley were bestowed “Honorary Member” status in recognition of their leading roles in establishing the field of neurocritical care.

## Conclusion

Evidence supports that receiving treatment in neurocritical care units significantly improves outcomes. In the future, as our understanding of disease pathophysiology deepens and new technologies are introduced, such as advanced imaging, monitoring systems, and the utilization of artificial intelligence, the care provided within neurocritical care units will only improve further. However, several ongoing challenges remain, including treating delayed cerebral ischemia in subarachnoid hemorrhage, refining surgical techniques after ICH, and discovering relevant neuroprotectants. To tackle these hurdles effectively, it is imperative to embrace innovative approaches. One promising approach is conducting “bundle research,” wherein combinations of various treatments and interventions are carefully studied to achieve better outcomes. Incorporating leadership and soft skills into patient care can significantly improve the overall treatment experience. Furthermore, instead of solely focusing on survival and functional outcomes, there should be a shift towards researching the quality of life and neurocognitive function of patients. This broader perspective will provide a more comprehensive understanding of the impacts of treatments and interventions on patients’ lives beyond just their physical recovery.

## Data Availability

Not Applicable.

## Abbreviations

ATACH-2: Antihypertensive Treatment of Acute Cerebral Hemorrhage-2
CCU: Coronary Care Unit
CLEAR-III: Clot Lysis Evaluation of Accelerated Resolution of Intraventricular Hemorrhage trial
CPR: Cardiopulmonary resuscitation
CSF: Cerebrospinal fluid
CT: Computer tomography
DGNI: Deutsche Gesellschaft für NeuroIntensiv- und Notfallmedizin
ECASS-4: ExTEND: European Cooperative Acute Stroke Study-4:extending the time for thrombolysis in emergency neurological deficits
EPITHET: Echoplanar Imaging Thrombolytic Evaluation Trial
ESETT: Established Status Epilepticus Treatment Trial
EXTEND: EXtending the time for Thrombolysis in Emergency Neurological Deficits
FOUR: Full Outline of UnResponsiveness score
GACHE: German trial on Aciclovir and Corticosteroids in Herpes-simplex-virus-Encephalitis
GBS: Guillain-Barré syndrome
GCS: Glasgow Coma Scale
GISSI: Gruppo Italiano per lo Studio della Streptokinasi nell’Infarto miocardico
GUSTO: Global Utilization of Streptokinase and Tissue Plasminogen Activator for Occluded Coronary Arteries trial
HSV: Herpes simplex viruses
ICES: Intraoperative Stereotactic Computed Tomography–guided Endoscopic Surgery for Brain Hemorrhage trial
ICP: Intracranial pressure
INTERACT-2: Intensive Blood Pressure Reduction in Acute Cerebral Hemorrhage Trial-2
IVIG: Intravenous immunoglobulin
MCA: Middle cerebral artery
MG: Myasthenia Gravis
MISTIE: Minimally Invasive Surgery plus Alteplase in Intracerebral Hemorrhage Evacuation trial
NCS: Neurocritical Care society
NMDAR: N-methyl-D-aspartate receptor
rt-PA: Recombinant tissue plasminogen activator
STICH: Surgical Treatment for Intracerebral Hemorrhage trials
TBI: Traumatic brain injury
TTM: Targeted Temperature Management

## Footnotes

1 Deutsche Gesellschaft für NeuroIntensiv- und Notfallmedizin.

2 Moniz was awarded the Nobel Prize in Physiology or Medicine in 1949 though this was for frontal lobotomy, the now abandoned surgical procedure once used as a psychiatric intervention.

3 EMI stood for Electric and Musical Industries and was the parent company of Parlophone, the German-British record company that signed the Beatles in 1962.

4 Hounsfield and Cormack were awarded the Nobel Prize in Physiology or Medicine in 1979.
